# A Sisterhood of Women: The Process of Co‐Creating Recommendations for Improved Pessary Self‐Management Support

**DOI:** 10.1111/jan.70387

**Published:** 2025-12-07

**Authors:** Lucy Dwyer, Sofia Tibuzzi, Kerrianne O'Rourke, Dawn Dowding, Rohna Kearney

**Affiliations:** ^1^ Division of Nursing, Midwifery and Social Work, School of Health Sciences, Faculty of Biology, Medicine and Health University of Manchester Manchester UK; ^2^ Saint Mary's Hospital Manchester University NHS Foundation Trust, Manchester Academic Health Science Centre Manchester UK; ^3^ Participant London UK; ^4^ Royal College of Obstetricians and Gynaecologists Women's Network London Bridge UK; ^5^ Institute of Human Development, Faculty of Medical & Human Sciences University of Manchester Manchester UK

**Keywords:** co‐creation, pessary, PPI, prolapse, self‐care, self‐management

## Abstract

**Aims:**

This work aimed to explore barriers to pessary self‐management and co‐create strategies to address these.

**Design:**

Participatory Action Research.

**Methods:**

In October 2024, eight pessary‐using women living in the United Kingdom participated in cooperative inquiry, discussion and co‐creation of strategies in two virtual workshops.

**Results:**

Pessary using women who participated in this research identified challenges affecting willingness to self‐manage a pessary and proposed solutions to address these and better support women. Pessary practitioners should assess physical capabilities, consider softer, more malleable pessaries, and explore the possibility of a pessary applicator. Peer support was seen as empowering, enabling self‐advocacy and improved care; therefore, establishing peer networks was prioritised. Major barriers included difficulty navigating services and limited access to a full range of pessaries, leading some women to buy devices online without medical oversight, creating a two‐tier system based on ability to pay. The group called for improved, standardised pessary care, and for self‐management to be reframed to avoid women feeling ‘fobbed off’ through better follow‐up, positive language, and compassionate care.

**Conclusions:**

The group identified strategies to address barriers to pessary self‐management which require further exploration. Pessary practitioners have a responsibility to listen to these voices and take steps to improve care for women in the future.

**Implications for the Profession and/or Patient Care:**

To support women's willingness to self‐manage their pessary, pessary practitioners should consider and support women to overcome physical and emotional barriers; improve information provision; maximise social support; boost women's perceived self‐efficacy; reframe pessary self‐management and ensure robust, accessible follow‐up is in place. This will ensure pessary‐using women are supported to make an informed decision about pessary self‐management. This research offers pessary practitioners insight into barriers women perceive to pessary self‐management and guidance as to how women can be supported to self‐manage their pessary.

**Impact:**

Only 21% of women are willing to self‐manage their pessary. Therefore, this research aimed to co‐create strategies to better support women to self‐manage their pessary and overcome barriers to willingness. Women reported individual, societal and service factors which affect willingness to self‐manage a pessary. These research findings should be translated into clinical practice and care delivery for pessary using women in both a community and hospital setting.

**Reporting Method:**

COREQ (COnsolidated criteria for REporting Qualitative research) Checklist.

**Patient or Public Contribution:**

Patients and members of the public were involved in research prioritization, study design, data analysis, interpretation of findings and dissemination.

**Study Registration:**

Study not registered.

## Summary Statement

**Why is this research or review needed?:**

○ Only 21% of pessary using women are willing to self‐manage their pessary for prolapse.
○ Women's unwillingness to self‐manage a pessary is influenced by fear of complications, perceived inability, low self‐efficacy, limited understanding of benefits, negative perceptions about self‐management and stigma.

**What are the key findings?:**

○ Individual, societal and service factors affect women's willingness to self‐manage a pessary for prolapse.
○ Practical strategies were created by women with lived experience of pessary use to enable other pessary using women to overcome barriers currently affecting willingness to self‐manage a pessary.
○ Well‐designed pessary self‐management support, informed by women with lived experience, may increase willingness to self‐manage a pessary.

**How should the findings be used to influence policy/practice/research/education?:**

○ Woman‐centred care, recognising the potential physical, psychosocial, and service‐level barriers to pessary self‐management will ensure care is equitable, responsive, and high quality.
○ Research exploring pessary applicator development, the physical requirements of pessary self‐management, peer support and whether certain pessaries facilitate self‐management should be prioritized.
○ Further research exploring pessary self‐management should be designed to ensure participants from diverse communities are enabled to participate and to ensure those with seldom‐heard voices are heard.

## 1 Introduction

Pelvic organ prolapse is a common condition caused by a weakness in the pelvic floor which significantly affects women's quality of life (Peinado Molina et al. 2023). Pessary management is an effective treatment that involves the insertion of a mechanical device into the vagina to provide structural support (van der Vaart et al. 2022). Pessaries are widely utilized; however the international scale of pessary management is poorly understood. Available evidence from the United Kingdom, the Netherlands and America suggests 87%–98% of clinicians offer pessary management to women with prolapse (Bugge et al. 2020). However, this evidence is limited to the three geographical regions, all being high‐income countries with well‐resourced healthcare systems (World Bank 2025; World Health Organization 2025). There is a wide range of types and sizes of pessaries available with limited evidence to guide pessary practitioners in the optimal type of pessary to use (Rantell et al. 2025). Support pessaries, made from polyvinyl chloride (PVC) or silicone are the most frequently utilized in UK practice (Brown et al. 2021). Pessaries made with silicone tend to be softer and more malleable than PVC pessaries; however they are also significantly more expensive (NHS Business Services Authority 2022). Internationally, guidelines recommend that pessary‐using women attend a face‐to‐face appointment with a pessary practitioner every 3–6 months unless they are self‐managing their pessary (Rantell et al. 2025). There is increasing evidence that pessary self‐management, whereby women are supported to remove and insert their pessary independently is both feasible and beneficial (Hagen et al. 2023). Other pessaries, such as the cube pessary, are routinely self‐managed (Nemeth et al. 2023). Despite this, only 21% of pessary‐using women are willing to self‐manage their pessary (Dwyer, Rajai, et al. 2024). Taking into consideration the widespread promotion of self‐management internationally (World Health Organization 2022), it is significant that pessary self‐management provokes such a strong negative reaction for so many women. This suggests the need for new approaches and informed intervention development, co‐designed by women with prolapse, to enable clinicians and the wider healthcare system to better support patients.

## 2 Background

Research exploring willingness to self‐manage a pessary has found that prior experience of, or fear of experiencing, pessary complications, perceiving oneself as physically unable to self‐manage, a lack of perceived self‐efficacy, not understanding the benefits of self‐management, perceiving self‐management negatively, as a lesser service, and the stigma and intimate nature of pessary self‐management, are factors that contribute to women feeling unwilling to self‐manage their pessary (Dwyer, Rajai, et al. 2024; Dwyer et al. 2022; Stairs et al. 2023; Storey et al. 2009; Börger et al. 2025; Kearney and Brown 2014). The cited research has been undertaken in the United Kingdom, the Netherlands and Canada, suggesting the barriers to self‐management identified are not isolated to a specific healthcare setting or culture, highlighting the international relevance of this work.

To address these barriers to pessary self‐management, it is necessary to target a range of behaviours; develop expertise and skill; and modify the intervention depending on context and the woman's individual needs. This meets the definition of a complex intervention according to The Medical Research Council (MRC) framework for developing and evaluating complex interventions (Skivington et al. 2021). The Index framework builds upon the MRC framework and suggests actions required to develop and evaluate complex interventions (O'Cathain et al. 2019). Two of these actions are evidence review and involving stakeholders (O'Cathain et al. 2019). Evidence review ensures that available evidence relevant to the intervention is considered and informs intervention development as appropriate (O'Cathain et al. 2019). Stakeholder involvement is essential to ensure the design of a pragmatic intervention which achieves what it aims to, and is perceived as beneficial by stakeholders (Skivington et al. 2021). Meaningful engagement with relevant stakeholders during intervention development has also been found to positively impact health outcomes and increase the likelihood of achieving change (Murray et al. 2010).

## 3 The Study

### 3.1 Aim

The aims of this research were firstly to use a collaborative approach to identify barriers that contribute to women's unwillingness to self‐manage a pessary for prolapse. The second aim was to co‐create strategies with pessary‐using women to develop an intervention to address barriers to pessary self‐management.

### 3.2 Study Design

Participatory action research (PAR) is a research approach which prioritises collaboration and power sharing with individuals with lived experience of the topic being researched, to ensure research outputs enable meaningful change and offer valuable new insight into a topic (Cornish et al. 2023). PAR describes the process of relevant stakeholders with lived experience of a problem, working together to create positive change (Kindon et al. 2007). Four key principles of PAR are valuing the participation of individuals with lived experience, knowledge in action, research as a transformative process and research through collaborative dialogue (Cornish et al. 2023). PAR is valuable where the aim of research is to solve complex problems with real‐world solutions (Vaughn and Jacquez 2020), therefore it is a valuable approach to intervention design and refinement. One type of PAR is co‐creation, which describes the exchange of knowledge, skills, ideas and experience between different stakeholders with the goal of meeting the needs of a population (Heimburg and Cluley 2021; Voorberg et al. 2015). Co‐creation provides a framework for ‘participative, collaborative, context‐sensitive and knowledge‐based practice that reflects the complex nature of health’ (Heimburg and Cluley 2021). The three‐stage framework for co‐production and prototyping health interventions developed by Hawkins et al. (2017) details the three stages necessary, including evidence review and stakeholder consultation, co‐production and prototyping. Co‐creation is therefore an appropriate methodology to consider the current challenges faced by pessary‐using women and to collaboratively develop strategies to overcome these informed by women with relevant lived experience.

### 3.3 Sample

The sample was eight pessary‐using women, purposively sampled from the Royal College of Obstetricians and Gynaecologists Women's Voices Involvement Panel. This is a group of over 600 women with personal experience of women's health issues, willing to be involved in research. Pessary‐using women from Women's Voices were deemed appropriate to participate because they had experience using a pessary. To maximise the breadth of conversation, we chose to recruit participants from a national group rather than a single hospital setting, to ensure they had experienced care from different healthcare organisations and therefore had varied experiences and views. We also purposefully recruited women of different ages and who used different types of pessaries to ensure a varied sample. It is recommended that focus groups have between five and 10 participants (Gray and Grove 2021), and that co‐creation research follow the same sample size principles as other focus group studies (Leask et al. 2019). Leask et al. (2019) suggest that for co‐creation research, a sample size of 10–12 co‐creators is advisable to account for attrition over time. Based upon this, the target sample size for the co‐creation focus group was 10. Unfortunately, despite repeated attempts by Women's Voices, additional women who were willing to participate were not identified. Therefore, a pragmatic decision was made to proceed with eight participants and to hold the sessions a week apart, in October 2024, to reduce the likelihood of attrition over time.

### 3.4 Recruitment

Regulatory approval was obtained from The Health Research Authority and Central Bristol Research Ethics Committee (Reference: 22/SW/0102). Standard text explaining the nature of the project and study participation was shared by Royal College of Obstetricians and Gynaecologists Women's Voices via social media and email distribution. The inclusion criteria required women to be female, willing and able to give informed consent, be over 18, have retained a pessary for prolapse for at least 2 weeks and be able to speak and understand English. Women who expressed an interest in participating were provided with written information about study participation and had a virtual one‐to‐one meeting with the researcher who discussed the study and obtained informed consent in accordance with Good Clinical Practice (Dixon 1998). Women received £110 for each co‐creation workshop attended in accordance with the National Institute for Health and Care Research public contributor payment policy (National Institute for Health and Care Research 2025). All women who expressed an interest in participating proceeded to take part in both workshops. To maintain participant confidentiality, each woman was assigned a pseudonym, which is used in conjunction with her verbatim quotations.

### 3.5 Data Collection

In accordance with Hawkins et al.'s (2017) co‐production framework, the aim for stage one was co‐operative inquiry via evidence review and stakeholder consultation with co‐creation participants. To facilitate this, in advance of the first workshop, participants were sent the findings of a scoping review of published evidence related to pessary self‐management, a systematic review of qualitative evidence exploring barriers and facilitators to self‐management of chronic conditions in women, a questionnaire study and qualitative findings exploring factors associated with willingness to self‐manage a pessary for prolapse (Dwyer, Rajai, et al. 2024; Dwyer et al. 2022; Dwyer, Barber, et al. 2024). Participants were asked to review this evidence, consider their own experiences and attend the workshops prepared to share their thoughts about pessary care and self‐management. In the co‐creation workshop, women were asked to use knowledge gained from the evidence review and their lived experience to make sense of and draw conclusions about factors which may affect willingness to self‐manage a pessary. The aim for stage two was for co‐creation participants to collectively identify strategies to address barriers to pessary self‐management willingness identified in stage one. Two 90‐min virtual meetings were held a week apart. These were supplemented by additional communications via email when participants wished to communicate with the researcher or facilitator outside of the group session to maintain privacy around subjects they wished to raise anonymously. The researcher is a nurse with a special interest in pessary management who was undertaking her PhD exploring factors that affect willingness to self‐manage a pessary for prolapse. This research was part of her PhD. To undertake this research the researcher undertook training in participatory action research as well as qualitative research methods training. Workshops were coordinated and facilitated by the lead for the RCOG Women's Network due to her experience in this role, to ensure equal participation between the researcher and stakeholders. The facilitator is an experienced patient and public involvement coordinator with extensive experience working within women's health. Demographic data, information about pessary use and self‐management experience was collected from each participant to enable reporting about participants. To ensure sentiments of co‐creation were followed, no topic guide was provided by the researcher. However, the facilitator was asked to review the same evidence provided to stakeholders and identify areas for discussion from this. The discussion prompts utilized are detailed in Table 1. Audio recordings of the co‐creation workshops were transcribed verbatim by a professional transcription service.

**TABLE 1:** Focus group discussion prompts.

What does pessary self‐management mean to you?
2 What are your experiences of pessary care?
3 What do you perceive are barriers to pessary self‐management?
4 What do you perceive are facilitators to pessary self‐management?
5 What are your thoughts about a pessary applicator as suggested in the evidence review?
6 What are your thoughts on the term pessary self‐management?
7 Do you have any specific reflections following the evidence review which we have not already discussed?
8 What is your preferred location for pessary care?
9 What healthcare professional support do you think is important for pessary using women and women self‐managing their pessary?

While the concept of data saturation is a commonly used principle in qualitative research (Gray and Grove 2021), it was not formally assessed in this study. This was a pragmatic decision, as the project was conducted within the scope of a PhD study with associated time and resource limitations. Instead, the number of focus groups was determined a priori based on what was feasible within the project timeframe. Therefore, the aim was not to reach theoretical saturation, which is in itself a challenging concept (Saunders et al. 2018), but to explore the range of perspectives within the participant group. Participants were purposively sampled to have relevant lived experience, but also to be articulate, well‐informed and communicative to ensure richness in the data (Gray and Grove 2021). Following analysis, the research team reviewed the findings and concluded there was sufficient evidence to achieve the research aims.

### 3.6 Analysis

The focus group transcripts were analysed thematically by the researcher in accordance with Braun and Clarke's six‐step process (Braun and Clarke 2006). The researcher started by familiarising herself with the data, listening to the audio recording and reading and re‐reading the transcripts to ensure a deep understanding of the discussion. After this, transcripts were read line‐by‐line and the researcher created codes that represented the content of that part of the discussion using NVivo software (version 14). The researcher then grouped codes into broader themes and subthemes when patterns within the data were identified. Themes and subthemes were named based upon the researcher's interpretation of the themes that had emerged. The themes were then reviewed by the researcher, a participant of the co‐creation workshop and the workshop facilitator, who performed synthesised member checking to ensure the analysis accurately represented the discussion and that names assigned to themes correctly represented their meaning.

### 3.7 Rigour

There are various methods to ensure rigor in qualitative research (Gray and Grove 2021). Firstly, to achieve reflexivity, researcher debriefing took place with the supervisory team (DD and RK), who discussed the findings and potential for bias based upon the researcher's positioning (Gray and Grove 2021). Hawkins et al.'s (2017) framework for co‐creation was followed to ensure the study was conducted in accordance with the principles of co‐creation. Respectful and trusting relationships were established between the researcher and participant by individual meetings before the focus groups to discuss the reasoning for this work and offer the opportunity to discuss the study on a one‐to‐one basis. Focus groups were audio recorded and transcribed to ensure accuracy. Validation was achieved via synthesized member checking by both the workshop facilitator and a participant (Gray and Grove 2021; Birt et al. 2016). The same participant and facilitator co‐authored this paper in accordance with the ethos of co‐creation, but also to prioritize the voice of those with lived experience. COREQ guidelines were followed in the reporting of this research to ensure transparency and quality in dissemination (Tong et al. 2007).

## 4 Findings

Eight women participated in the co‐creation process, as well as the female facilitator and female researcher. The mean age of participants was 53 years (range: 38–71). All participants were White, with most being White British and the other women White Irish and White European. Six participants were educated to college or university level or above, three having post‐graduate degrees. Five women used cube pessaries, which must be self‐managed as per manufacturer instructions. Two women used silicone rings and one a donut pessary. All participants self‐managed their pessary. Themes from the co‐creation workshop were physical barriers, knowledge and understanding, social support, access to pessary services, emotional barriers perceived self‐efficacy and the perception of self‐management.

### 4.1 Cooperative Inquiry—Physical Barriers

Women reported times when they had found pessary insertion or removal painful, meaning they limited pessary use to avoid self‐management, describing it as an ‘absolute downer’. The physical requirements of pessary self‐management mean women must be able to compress their pessary and maintain this compression during insertion and removal. Therefore, this was raised as a potential barrier to pessary self‐management. While everyone was currently able to self‐manage, they were conscious aging may impact their manual dexterity:

I don't know how women who have less strength than me, or who perhaps have things like arthritis, or issues with manual dexterity…I have absolutely no idea how they would even begin to try and insert this … I don't struggle…at my current age…but I do worry that as I get older and as my dexterity does decline slightly that I would also struggle with that.—Anna

Women found a way to overcome the physical challenges of self‐management by using ‘soft squishy’ pessaries which were easier to self‐insert and remove and perceived as more comfortable. However, as silicone pessaries are more expensive, they are restricted by some organisations. To address this, some of the participants had navigated this by buying their own pessaries, because, ‘what else are we supposed to do’? Another woman had been advised to buy her own pessary by her doctor, but hadn't, because she couldn't afford to:

there's two systems: there's the NHS, which provides a standard set of pessaries, they are universally cheap and nasty and uncomfortable, and mostly don't work. And then if you go outside of the NHS there's a whole load of nice, soft, squishy silicone ones, and different designs, and different kinds that the NHS can't or won't get. And women are self‐funding and going outside of the NHS to buy something off their own back, with their own money.—Alice

Several women discussed procuring or planning to procure a specific pessary which, on investigation, is not defined as a medical device but a consumer product and therefore has not undergone the same rigorous safety, quality and clinical efficacy testing as vaginal pessaries. Women also reported self‐managing pessaries they had procured online without medical oversight. One woman reported experiencing vaginal bleeding semi‐regularly, but because she was unsure what to do and when to be concerned, ‘puts it back and hopes for the best’.

### 4.2 Co‐Created Strategies‐Physical Barriers

To address the physical requirements of self‐management, the group was asked to discuss the concept of a pessary applicator as proposed in the evidence review. The idea was well received, with women feeling it would be ‘helpful’ and ‘make (self‐management) easier.’ Suggested functionality of a proposed pessary applicator was that it should:

squish the pessary for you, and then you could release it when it was in the right place—Nicole.

Because disparities in access to certain types of pessaries may be a barrier to self‐management initiating or continuing, it was proposed that access to a wide range of pessaries should be standardised, regardless of a woman's geographical location, local contracts and financial arrangements, via creation of a national pessary repository. One suggestion to standardise access to pessaries was to develop evidence‐based national guidelines:

I've been thinking since we spoke last week that this lack of standardisation and so on…it seems essential that there is uniformity…among GPs, among gynaecologists, and among pessary nurses. And I think the only way that that can be achieved is if we had a NICE guideline for the management of pessaries.—Caroline

### 4.3 Cooperative Inquiry‐Knowledge and Understanding

The importance of being knowledgeable about and understanding self‐management and pessary management was repeatedly discussed by women. Many had independently researched prolapse, different management options, self‐management and pessary types to empower them to advocate for themselves when interacting with healthcare professionals. When asked what makes self‐management easier, one woman replied:

For me it's having the knowledge, and I've gained that knowledge either from my own self research, or mainly from the Facebook support group, so from peer support, from other people sharing their stories, from other people putting up advice of potential new pessaries to try, or how to even insert and remove them. It's all been from those support groups, it's not been from my NHS provider, and that's what's made my journey so much easier.—Anna

Participants felt it necessary to have this knowledge and understanding to ensure they could self‐advocate and make informed decisions regarding pessary care and self‐management:

I did my own research and found that the cube was the only one that really would work for me, according to the literature. And when I went back to the urogynaecologist I asked for a cube.—Alice

Cube pessaries require daily removal. Despite this, women using cube pessaries reported being given no information or guidance about how to self‐manage their pessary. It was suggested this may discourage some women from self‐managing as they wouldn't know what to do:

I was just fitted with the cube and sent away, and ‘oh well, you're self‐managing now, so now you're off the list’. So I didn't have any follow up, I didn't have any instruction, I didn't have anywhere where I could turn to if I was really struggling to get the damn thing out, which I was.—Alice

A further issue regarding information provision was the receipt of conflicting information, such as the types of pessaries which could be self‐managed. This resulted in women losing confidence in the advice given:

the lack of continuity of information I found quite astonishing from different providers, of being told one thing one time, and then the exact opposite by somebody else, extremely confidently [laughs]… they just seem to all have completely different information, which is quite frustrating—Leanne

One suggestion was that, as with medication, there should be a requirement for written information to be provided to service‐users as standard when using a pessary which must be self‐managed:

Why is there not a leaflet that comes in the box that the cube is given out with, that tells you exactly what to do?—Alice

Pessaries tend to be supplied in packaging with an information sheet enclosed, therefore it appears instead of providing these to women who are going to self‐manage, instead the leaflets are automatically being discarded.

Specific knowledge gaps highlighted by women related to self‐management, were what to do if a pessary is expelled, what to do if experiencing pessary complications and cleaning and storage instructions. The importance of written information to refer to and visual images was also highlighted by participants:

I think everyone can appreciate that when you're in these intimate meetings with your urogynaecologist your brain's not truly functioning to take everything in that they're telling you. So to just provide something like a leaflet of how to self‐manage afterwards, how to remove, how to insert, how to clean, all these sorts of things that we've mentioned, or even an online QR code somewhere that you can go find that information.—Alice

### 4.4 Co‐Created Strategies‐Knowledge and Understanding

To facilitate women to be knowledgeable and understanding, participants prioritised the development of high‐quality information which includes clear guidance and images, specifically detailing: pessary insertion; removal; cleaning; storage; and pessary complications. It was also proposed that information resources should be developed and shared online to enable wider access to trustworthy, evidence‐based information.

### 4.5 Cooperative Inquiry‐Social Support

The importance of social support for women with prolapse was highlighted. Participants discussed the emotional support they'd received from friends with similar lived experiences and supportive spouses.

I have three close friends who also have pelvic organ prolapse, so we do share our experiences with each other, we support each other.—Kirsty

Women agreed that self‐management gets easier with practice, and they needed practical support from their partner facilitating them to have time to learn, relax and practice. Participants stated that without emotional and practical support they received, they wouldn't be able to self‐manage.

I couldn't have done without, and I think if I didn't have the support of my partner, I don't think I would have psychologically been able to self‐manage as well.—Anna

Due to the taboo nature of prolapse and pessaries, other women relied upon social support from peers via online forums:

I can't tell any of my friends that I've got pelvic organ prolapse, I'm too embarrassed, I can't talk to anyone about it other than online.—Alice

For these women, online peer support was invaluable. As well as information, women described the ‘camaraderie’ between pessary using women which was extremely meaningful to them:

I think the one word that would sum up my experience…is ‘sisterhood’, that I have learnt so much from other women.—Caroline

### 4.6 Co‐Created Strategies‐Social Support

In terms of real‐life social support, the only suggestion from the workshop to increase this, was to destigmatize and raise public awareness of prolapse, which in turn would result in friends and family having a better understanding of, and therefore knowing how best to support women:

Then it would be easier maybe even for your partners at home, because it's not something that is so random and they have never heard about before. Because if it's something that we all talk about it would help all round I think, so I think it really comes a lot to that.—Sarah

A specific co‐created solution was to establish peer support networks for women with prolapse and pessary using women. Participants believed many women desired and would benefit from peer support. It was suggested peer support should be available both online and face‐to‐face, to ensure accessibility to everyone. Participants recognised peer support groups would need to be moderated as well as having a clear patient‐focused aim.

### 4.7 Cooperative Inquiry‐Access to Pessary Services

Long waiting lists which caused delays accessing care, created feelings of frustration for women, including a recently retired healthcare professional:

I was quite annoyed with myself at not being able to navigate the NHS—Caroline

Furthermore, once women had overcome gatekeeping, women perceived there was a ‘lottery’ regarding care and that you were ‘lucky’ if you lived locally to a good service:

even to get to the right pessary you had to find the right person…I was lucky to.

But it's a bit of a gamble isn't it‐Sarah.

Due to these challenges navigating and accessing pessary care, women were reticent about the concept of self‐management and being ‘off the books’, because they perceived it as leaving the security of clinical care and putting them at risk of having to repeat a difficult referral process or losing a trusted pessary practitioner.

Participants reported their experience of inconsistent levels of follow‐up for self‐managing women. Some were discharged completely, some discharged to primary care and others continued to receive intermittent follow‐up from a dedicated pessary service. Participants believed regular ongoing routine follow‐up should continue for self‐managing women. Ongoing care ensured women knew they were going to be seen by a definitive time point, offered safety netting in case prolapse symptoms got worse and reassured women they were still part of a service and therefore could contact the team if experiencing issues.

Because some women had negative experiences when initially seeking treatment for prolapse in primary care, they lacked confidence in their GP's knowledge and skill related to prolapse and pessary care. Therefore, despite receiving inconsistent pessary care in secondary care, women still preferred to attend a less convenient hospital for follow‐up:

for me the GP I never really feel that I'm in such good hands unfortunately, I feel like I need to get past that, and as soon as I get to that I've had a really good mix, but also very good care at the hospital. So kind of I guess emotionally it feels better to go there [laughs], because I feel like I'm at the right place, they know a bit more kind of thing.—Sarah

Furthermore, for self‐managing women automatically discharged from a pessary service in secondary care to primary care, the worry of having to rely on GPs in the instance of pessary complications or the need for support was identified as a barrier to self‐management.

I worry that now I've essentially been discharged from the urogynae service that when my prolapse gets worse, that I have to then go through that awful rigmarole of GP referral and things like that.—Anna

Another woman didn't understand the value of follow‐up with the GP as the importance of regular vaginal examinations for pessary‐using women had not been sufficiently explained, causing the woman to disengage with the service:

They'd never even told me that I'd been put on a pathway to go to my GP every six months. So by the time I turned up there, I said ‘well I've been managing it myself for the last five months, what's the point in coming to see you?—Alice

Women also reported a disconnect between pessary care in secondary and primary care. One example was a woman being discharged from secondary care for pessary follow‐up with her GP. However, the GP couldn't provide her with replacement pessaries, which resulted in her being referred back to secondary care and a lengthy wait for a new pessary.

### 4.8 Co‐Created Strategies—Access to Pessary Services

There were no specific strategies proposed to address participants' dissatisfaction and frustration with the complexities of accessing prolapse and pessary care, which in turn may cause unwillingness to self‐manage a pessary. This may be because women's comments suggested they were pessimistic about the reality of current health services:

because it's not an ideal world, the way I feel about the healthcare system—Sarah

In terms of ongoing follow‐up for self‐managing women, participants felt self‐managing women should have the option of long‐term scheduled follow‐up from a specialist pessary service. It was felt that pessary follow‐up appointments should be face‐to‐face rather than via telephone, partly due to the importance of regular vaginal examinations but also to facilitate a connection between the woman and pessary practitioner. In addition to routine follow‐up, participants also prioritised continual accessible support from a pessary practitioner for all self‐managing women:

I have a bit of an awareness about other areas that have things such as specialist nurses. And I think something like a specialist pessary nurse, I'm not saying someone who would be on call 24/7…but just somebody who would be available via a work phone or a work email address for those little questions that you have every now and again.—Anna

### 4.9 Cooperative Inquiry‐Emotional Barriers

It was felt by participants that many healthcare professionals dealing with prolapse lacked compassion, did not understand the devastating emotional impact of prolapse, the embarrassment women feel, and their vulnerability. Pessary appointments often felt ‘rushed’; therefore the psychosocial impact of prolapse and pessary use was not discussed. Conversely, women did not feel this way about care from a women's health specialist physiotherapist. Leanne felt the physiotherapist she saw was:

the only one who was really sympathetic, and kind, and took on board that this was a really traumatic thing that had happened.

Women also felt physiotherapists gave them hope their symptoms could improve and worked with them to achieve goals such as running again:

the physio was the one who gave me more hope I think, in terms of that my life wasn't over—Sarah

The emotional impact of having a diagnosis of prolapse was raised as a factor affecting willingness to self‐manage. Women described how prolapse affected their identity, which was particularly impactful for those who'd developed symptoms at a younger age, or where it impacted upon their ability to be physically active. Participants also discussed the common misperception that prolapse was something which happened solely to older women, with the consensus that the ‘younger you are, the worse it must be’.

Women also described how frightening developing prolapse and commencing self‐management was, suggesting a recent diagnosis of prolapse or being new to pessary management may make women less willing to self‐manage due to fear:

I think in the first year or so I would have not been open to it, because I was still adjusting to the whole thing, and I wanted contact with professionals, and I was quite scared about a lot of it. But when you're a bit further along the journey then I think you're a bit more open to it. So I guess if women aren't up for it straight away when they've just got a prolapse, they might be a year or two in I guess.—Leanne

Women shared that being diagnosed with prolapse had negatively impacted on their identity and that self‐management of pessaries, particularly those which need to be removed daily, was a ‘constant reminder of having a problem’ which detrimentally affected their mental health and may affect willingness of others to self‐manage:

having to insert and remove every morning and every night, it's the first thing I think of in the morning and it's the last thing I think of at night, so it's got such a psychological barrier towards it.—Anna

### 4.10 Co‐Created Strategies‐Emotional Barriers

Participants suggested supported self‐management requires pessary practitioners to understand and consider the emotional impact of prolapse diagnosis and subsequent treatment and respond positively while acknowledging the woman's feelings. Furthermore, because women's feelings about pessary self‐management may change over time, the opportunity to self‐manage should not be a one‐off discussion.

### 4.11 Cooperative Inquiry‐Perceived Self‐Efficacy

During the workshop, participants used phrases alluding to their perceived lack of choice or options regarding pessaries or self‐management:

I was sort of forced into having to self‐manage. And it's taken me, psychologically, a really long time to almost see the benefits of that—Anna

and

the cube was the only pessary that would manage my symptoms, the only one that was suitable. So I had no option but to self‐manage—Alice

The feeling of having ‘succumbed’ to pessary management, or the use of a particular pessary, rather than feeling they had a choice in decision making and were receiving an effective, beneficial treatment, may negatively impact upon women's beliefs about pessary management via poor outcome expectancy and therefore willingness to, and acceptability of, self‐management.

Conversely participants alluded to their perceived self‐efficacy in terms of bodily awareness, decision making regarding pessary use and the ease of pessary self‐management:

(Self‐management) ‘does get easier with practice’—Nicole

and

if I'm going to sit at home and do lots of paperwork or just do small bits of housework, I prefer to give my body a rest…So I manage my pessary route around my day, and I prefer to have several days a week, if possible, when I'm not using it. But I'm then aware that things are dropping lower and lower and it's getting unmanageable, so I have to put the pessary back in again—Alice

As well as daily activities, women gave examples of factors which influenced their decision‐making about pessary use, which included how their body felt, urinary symptoms and giving their body a rest.

Interestingly, women who made statements about their perceived lack of choice in decision‐making about pessary management or self‐management, also gave examples of perceived self‐efficacy. This demonstrates some participants were able to overcome feelings of lacking autonomy to regain control of their pessary self‐management.

Participants also gave examples of self‐advocating for elements of pessary care as a further example of self‐efficacy. Women described the struggle to ‘get my voice heard’ by healthcare professionals and that they had to ‘push’ to receive elements of pessary care such as a prescription for topical oestrogen or a specific pessary.

‘I think you have to be articulate…and self‐advocate and experiment and find out what works for you.’—Caroline

### 4.12 Co‐Created Strategies‐Perceived Self‐Efficacy

All the participants self‐managed their pessary and most demonstrated high levels of perceived self‐efficacy. Women stated that knowledge and ability to self‐advocate was essential for pessary‐using women and specifically self‐managing women to have confidence in self‐management. Therefore, to boost perceived self‐efficacy, self‐management support should include strategies to ensure women receive information which facilitates self‐advocacy, as well as being encouraged to be a partner, or director in care and decision‐making and to self‐advocate. To prevent women from perceiving pessaries as a lesser option than surgical management, the value and benefits of pessary management compared with surgical management, should be discussed and women encouraged to be active partners in decision‐making about whether they wish to use a pessary. Women gave examples of different ways in which they self‐managed their pessary to meet their individual needs. The ability to self‐determine pessary use to manage prolapse symptoms was cited as a key benefit of self‐management by participants. Therefore, women should be empowered to use their bodily knowledge and experience to do this.

### 4.13 Cooperative Inquiry‐Perception of Self‐Management

Women highlighted after waiting so long to see a clinician, self‐management could be viewed negatively, as being ‘fobbed off’.

(healthcare professionals) want this (self‐management) to happen so we can ditch you off the system [laughs] and go ‘you can sort this out yourself’, which I think women feel they are expected to do in so many areas of life.—Leanne

The same woman became so disillusioned with her pessary care, she perceived self‐management to be:

something that I just initiated myself through frustration with lack of care and continuity of care really.—Leanne

These motivations demonstrate self‐management can be viewed as something women are forced to do, or as an unhappy necessity, rather than as a positive opportunity to become an empowered partner in pessary care with potential benefits for the woman.

### 4.14 Co‐Created Strategies—Perception of Self‐Management

It was agreed by participants there was a need to reframe pessary self‐management as a positive thing for women, not just a way of offloading patients. Suggestions to achieve this were emphasising the benefits of self‐management, specifically that self‐management offered women control over their pessary and what goes into their body which was highlighted as being important:

maybe it is about control, to not be like you're sent home to do your thing, but more like to put it in a way that you can control your own POP journey or whatever, control that so it becomes something positive that you take charge.—Sarah

Women also perceived that self‐management facilitated increased confidence and flexibility in how they used their pessary.

Evidence reviewed in stage one suggested the need to consider whether the term ‘self‐management’ incorrectly suggests complete independence and a lack of oversight and support and therefore discourages women from self‐management. When prompted to discuss this, a participant commented:

I think that makes a lot of sense…if you've got those two different ones, the clinic led, the patient led, then yes, it might give you that sense of ‘okay, this is me’, that you still have the support. Because I think yes, that is a good thing that you have the combination, some support, but you take the control.—Sarah

Conversely, woman disagreed with ‘patient‐led care’ because:

we're not sick, we're not patients. We're healthy women, self‐advocating for better health.—Caroline

The suggestion of ‘woman‐led care’ was better received as an alternative to self‐management.

The need to consider possible and reasonable adjustments to self‐management support for women with neurodiversity was highlighted as an important consideration.

## 5 Discussion

Participants in the co‐creation workshops undertook an evidence review and used their lived experience to collaboratively identify barriers to pessary self‐management, including physical requirements, knowledge and understanding, social support, access to pessary services, emotional barriers, perceived self‐efficacy and perception of pessary self‐management. The women then co‐created strategies to overcome these barriers (Table A1).

Women valued access to a wide range of pessaries, particularly softer, more malleable types and felt this was crucial to enabling self‐management. However, limited NHS availability led some women to privately procure pessaries, creating inequities between those who could and could not afford this. In one case a woman had purchased a non‐regulated consumer device, marketed as a pessary, leaving her vulnerable to complications. These findings highlight the need for evidence on the clinical and cost‐effectiveness of different pessary types and for an evidence‐based guideline to support equitable, safe provision across services. A pessary applicator was considered by participants to be a potentially valuable way to navigate physical barriers to pessary insertion, with international evidence suggesting this is a device that is desired by some pessary‐using women (Mussawar et al. 2024). High‐quality written information was felt to be essential but currently lacking. While the UK pessary guidelines include written patient information that can be provided to women, participants were unaware of the guidelines, and none had received the written information. Therefore, raising awareness of existing guidelines and available resources among pessary practitioners should be prioritized. The need for National Institute for Health and Care Excellence (NICE) guidelines for pessary care was discussed by participants. Existing NICE guidelines regarding prolapse management make reference to the impact of pessaries on sexual intercourse, potential pessary complications and follow‐up requirements for pessary‐using women (National Institute for Health and Care Excellence 2019). However, this co‐creation study identified that current guideline recommendations do not offer the level of detail desired by participants. Therefore, there is a need for pessary‐specific guidelines to be commissioned, particularly taking into consideration the scale of pessary use in the UK (Dwyer et al. 2021). Peer support, both online and face‐to‐face, was perceived as highly valuable, offering social and emotional benefits in addition to clinical ones. Evidence from other long‐term conditions suggests such networks can be feasible and effective (Thompson et al. 2022), but their role in pessary care is not yet clear and requires further exploration. Participants expressed dissatisfaction with aspects of pessary care, particularly the lack of compassionate, person‐centered interactions. Experiences of disempowerment and paternalism from GPs, Urogynaecologists and pessary nurses contrasted sharply with more positive accounts of care from physiotherapists, who were viewed as supportive, holistic, and empowering. Women valued care that acknowledged the psychosocial impact of prolapse, respected their embodied knowledge, and worked in partnership with them to achieve personal goals. To support this, further work is needed to embed compassionate, holistic, and collaborative approaches into pessary services, including exploring the impact of different appointment lengths, what the necessary constituents of pessary care are, and understanding what pessary‐using women prioritize at these appointments.

This co‐creation study highlighted that there are multiple factors which influence willingness to self‐manage a pessary for prolapse. Our findings echo those of other qualitative work exploring pessary self‐management. In this study, concerns about physical ability to insert and remove a pessary were reported. Physical barriers are a frequently reported barrier to pessary self‐management (Stairs et al. 2023; Kearney and Brown 2014; Roberts et al. 2024). Furthermore, in Börger et al.'s (2025) research, women with physical concerns about their ability to self‐manage had poor outcome expectancy and were therefore unwilling to attempt self‐management. Despite this common finding, the physical requirements for pessary self‐management have not yet been established.

Participants identified healthcare professional support as being a major factor impacting willingness to self‐manage a pessary. Börger et al. (2025) reported women valued pessary practitioner guidance as part of self‐management support. Furthermore, a close bond between pessary practitioners and pessary‐using women was identified by Storey et al. (2009), with women reporting psychological and emotional support. However, it was unclear whether this support increased women's willingness to self‐manage, or conversely, whether the desire to continue attending appointments and receiving this support deterred women from self‐managing.

Knowledge is necessary for self‐management because it enables the decision making required (Lorig and Holman 2003). Despite this, co‐creation participants reported inadequate information provision. This was also a finding in other studies whereby women reported their perception that they lacked the knowledge required to self‐manage a pessary (Stairs et al. 2023; Roberts et al. 2024). Women's fear of inserting the pessary incorrectly was reported by Börger et al. (2025). However, the same women emphasized this worry could be easily addressed by pessary practitioners via information provision (Börger et al. 2025). Informational needs identified in other studies were practical instructions on pessary insertion and removal and how to navigate sexual activity alongside pessary management (Börger et al. 2025; Roberts et al. 2024).

Self‐efficacy theory is the concept that an individual's belief they can perform a behaviour impacts the likelihood they will achieve it (Bandura and Adams 1977). Therefore, self‐efficacy is known to be a facilitator of self‐management (Dwyer, Barber, et al. 2024). Co‐creation participants shared their belief that perceived self‐efficacy impacts women's willingness to self‐manage their pessary, and thus to maximise this, self‐management support should include strategies to boost self‐efficacy. This mirrors findings in other similar studies whereby the importance of women having confidence in their ability to perform self‐management was emphasised (Stairs et al. 2023; Storey et al. 2009; Börger et al. 2025; Roberts et al. 2024).

A key finding of this co‐creation study is dissatisfaction with current prolapse and pessary care reported by women. Participants suggested this was another factor that may reduce women's willingness to self‐manage their pessary. This was not an expected finding; literature about pessary clinics tends to suggest women experience positive, supportive care (Storey et al. 2009; Aston and Storey 2023). In fact it has been suggested that some women choose not to self‐manage their pessary because of the value they place on attending pessary clinics (Storey et al. 2009). However, as with the findings of this study where women described feeling disempowered and vulnerable when receiving pessary care, Abhyankar et al. (2019) also reported women experiencing ‘traditional, paternalistic’ interactions when seeking treatment for their prolapse. Interestingly, in both studies, women's health physiotherapists were viewed favourably as knowledgeable and supportive, creating feelings of ‘hope, empowerment and self‐efficacy’ (Abhyankar et al. 2019). Furthermore, in another study participants perceived physiotherapists as being the only healthcare professionals who acknowledged the significant impact prolapse had on their life and worked alongside them to overcome this (Carroll et al. 2022). Abhyankar et al. (2019) suggest a key difference is that physiotherapists value women's embodied knowledge of their prolapse, and that this matters to women. It is important to recognise that the time for physiotherapy appointments tends to be longer than appointments in pessary clinics (Abhyankar et al. 2019). Furthermore, several participants in our study had paid privately to see a physiotherapist. Ultimately, it appears women value pessary care that meets their individual needs and where the woman is working in partnership with the pessary practitioner to achieve their desired goal (Abhyankar et al. 2019; Carroll et al. 2022).

## 6 Strengths and Limitations

Methodological strengths of this work are the use of a framework to guide the process of co‐creation (Hawkins et al. 2017), strategies to ensure power sharing such as facilitating an evidence review, use of an independent, experienced facilitator, and co‐analysis and interpretation of data by the researcher, workshop facilitator and a pessary‐using woman who participated in the co‐creation workshop. It is important to acknowledge the researcher was a participant in the workshops and her clinical experience and advocacy for pessary self‐management may have influenced the group discussion. To mitigate this, the researcher limited her contributions to those when asked a direct question by a participant, or if clarification from a clinical or research perspective was needed.

We aimed for co‐creation participants to be a varied sample to maximise breadth of conversation and views. However, all participants were white women, who performed self‐management; therefore this may limit the generalisability of findings. There is a paucity of evidence about pessary self‐management in women from ethnic minority/global majority backgrounds. However, available evidence suggests women from a Hispanic background have a similar pessary management continuation rate as women from other ethnicities (Roberts et al. 2024) despite prior knowledge regarding pessaries being poor (Maldonado et al. 2021). Furthermore most of the Hispanic women in one study became comfortable and confident to self‐manage their pessary (Maldonado et al. 2021). All of the participants completed further education, with most of the women (75%, *n* = 6) being educated to degree level or above. Further work to engage with women with different levels of educational attainment and from different ethnicities is necessary to ensure any interventions developed are suitable for all women. The sample was self‐selected. Therefore, those who have positive or negative experiences with pessary care may be more motivated to participate and share their stories. However, other recent work highlights similar experiences, suggesting this is part of a wider system problem (Abhyankar et al. 2019; Carroll et al. 2023).

## 7 Conclusion

Pessary‐using women are influenced by personal, physical, and psychosocial factors when considering pessary self‐management. The strategies co‐created by pessary‐using women demonstrate influential factors upon women's willingness and suggest ways of overcoming these barriers. Our findings also emphasise the importance of embedding holistic, collaborative approaches which value women's embodied knowledge into pessary care. Future research should address evidence gaps identified in this study. Firstly, regarding the clinical and cost‐effectiveness of different pessary types, alongside exploring the development of a pessary applicator. Further work is also required to develop and evaluate high‐quality written information informed by the views of pessary‐using women. Research should also be undertaken to explore how best to provide compassionate, holistic, and woman‐centred care, including optimal appointment length and aspects of care prioritised by women. The role of peer support networks, both online and face‐to‐face also requires exploration. Further work is necessary to establish the physical requirements of pessary self‐management as a frequently reported barrier. Furthermore, as pessary practitioners were not involved in this co‐creation study, it is essential to include them in future work to ensure cognitive participation, coherence and collective action from relevant stakeholders (Murray et al. 2010). Finally, future research should prioritise involvement and engagement with women with seldom‐heard voices due to the lack of representation in this study.

## Funding

This work was supported by the National Institute of Health and Care Research (NIHR300519).

## Conflicts of Interest

Lucy Dwyer was in receipt of a Clinical Doctoral Research Fellowship (NIHR300519) funded by Health Education England (HEE) and National Institute for Health Research (NIHR) for this research project. Lucy was also a co‐applicant of the NIHR/HTA funded Treatment of Prolapse with Self‐Care Pessary (TOPSY) study. Sofia Tibuzzi, Kerrianne O'Rourke and Dawn Dowding declare they have no conflicts of interest. Rohna Kearney was clinical chief investigator and co‐applicant of the NIHR/HTA funded Treatment of Prolapse with Self‐Care Pessary (TOPSY) study.

## Supporting information


**Data S1:** jan70387‐sup‐0001‐supinfo.pdf.

## Data Availability

The data that support the findings of this study are available from the corresponding author upon reasonable request.

## Appendix A

**TABLE A1:** Co‐created strategies to support pessary self‐management.

Factor affecting self‐management willingness	Co‐created strategies
1. Physical requirements	Pessary applicator Wide access to soft pessaries
2. Knowledge and understanding	High‐quality written information
3. Social support	Increased public awareness of pelvic organ prolapse and pessary management Establishing online and face‐to‐face peer support networks for women with pelvic organ prolapse and women who use pessaries
4. Access to pessary services	Continued long‐term follow‐up from a specialist pessary service for self‐managing women Accessible support from a pessary practitioner
5. Emotional barriers	Self‐management support to include discussion about, and support for, the emotional impact of developing prolapse and commencing pessary self‐management
6. Perceived self‐efficacy	Improve women's self‐efficacy Facilitate self‐advocacy Reinforce the value and benefits of pessary management of prolapse Empower women to use bodily knowledge and experience to self‐determine pessary use
7. Perception of pessary self‐management	Reframe pessary self‐management as a positive option Emphasise potential benefits Undertake work to explore rebranding pessary self‐management starting with a new name, possibly woman‐led car
